# Recycling and Degradation of Polyamides

**DOI:** 10.3390/molecules29081742

**Published:** 2024-04-11

**Authors:** Lin Zheng, Mengjin Wang, Yaoqin Li, Yan Xiong, Chonggang Wu

**Affiliations:** 1 Hubei Provincial Key Laboratory of Green Materials for Light Industry, Collaborative Innovation Center of Green Light-Weight Materials and Processing, New Materials and Green Manufacturing Talent Introduction and Innovation Demonstration Base, School of Materials and Chemical Engineering, Hubei University of Technology, Wuhan 430068, China; 102210475@hbut.edu.cn (L.Z.); mjwang@hbut.edu.cn (M.W.); yqli@hbut.edu.cn (Y.L.); xiongyan1980@hotmail.com (Y.X.); 2 Hubei Longzhong Laboratory, Xiangyang 441000, China

**Keywords:** polyamides, recycling, degradations, reaction mechanisms

## Abstract

As one of the five major engineering plastics, polyamide brings many benefits to humans in the fields of transportation, clothing, entertainment, health, and more. However, as the production of polyamide increases year by year, the pollution problems it causes are becoming increasingly severe. This article reviews the current recycling and treatment processes of polyamide, such as chemical, mechanical, and energy recovery, and degradation methods such as thermal oxidation, photooxidation, enzyme degradation, etc. Starting from the synthesis mechanism of polyamide, it discusses the advantages and disadvantages of different treatment methods of polyamide to obtain more environmentally friendly and economical treatment schemes. Finding enzymes that can degrade high-molecular-weight polyamides, exploring the recovery of polyamides under mild conditions, synthesizing environmentally degradable polyamides through copolymerization or molecular design, and finally preparing degradable bio-based polyamides may be the destination of polyamide.

## 1. Introduction

Polyamides (PA) are polymers that contain repeating amide groups (–CO–NH–) as part of the polymer main chain. The main chain of polyamide molecules contains amide groups, which have the characteristics of high mechanical strength, high rigidity, wear resistance, strong reinforcement, and impact resistance, and are among the five major engineering plastics [1]. Polyamide is insoluble in common solvents. The strong polarity of the amide group gives it a high degree of crystallinity and strength [2,3].

**Figure 1:**
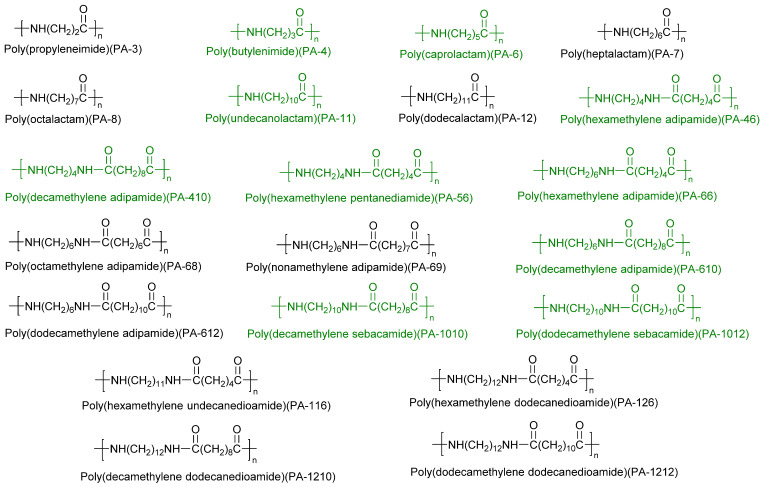
Common aliphatic polyamides. Commonly commercialized bio-based polyamides are highlighted in green.

There are several families of PA, which can be aliphatic, semi-aromatic, or aromatic, depending on the nature of the linkers separating amide functions. Nylon is the general term for aliphatic polyamide resins. Common aliphatic polyamides are shown in Figure 1. The synthesis methods of aliphatic polyamides generally adopt salt condensation, melt condensation, and solid-phase condensation. Due to the simple molecular structure, aliphatic polyamides have high crystallinity, fast crystallization speed, high melting point, and high density. Their good adhesion, flexibility, and thermoplasticity make them widely used in hot melt adhesives and plastic printing inks [1]. At present, most of the raw materials for polyamides are synthesized from petrochemical resources. With the increasing depletion of petrochemical resources, the research on bio-based polyamides is becoming more and more important [4,5]. The production cost of bio-based aromatic monomers is high, and the performance of bio-based aromatic polyamides still has a certain gap compared with traditional petroleum-based aromatic polyamides. The bio-based polyamides currently produced industrially are still limited to aliphatic polyamides. Generally speaking, if the source of polymer monomers contains materials derived from biomass or (and) obtained through biomanufacturing, it can be called bio-based polyamide. Fortunately, with the development of metabolic engineering and biocatalysis, more and more raw materials can come from biology [6,7]. Commonly commercialized bio-based polyamides include PA-11, PA-1010, PA-46, PA-56, PA-4, PA-6, PA-610, PA-410, PA-1012, PA10T, PA-66, etc. Although polyamides can be synthesized and produced through bio-based monomer raw materials, this does not guarantee their biodegradability [8]. At present, only PA-4 and itaconic acid-derived PA have been reported as biodegradable polyamides. The commercialized bio-based PA has a low market share due to the small number of production companies, and the output is less than 1% of the total output of PA [9].

**Figure 2:**
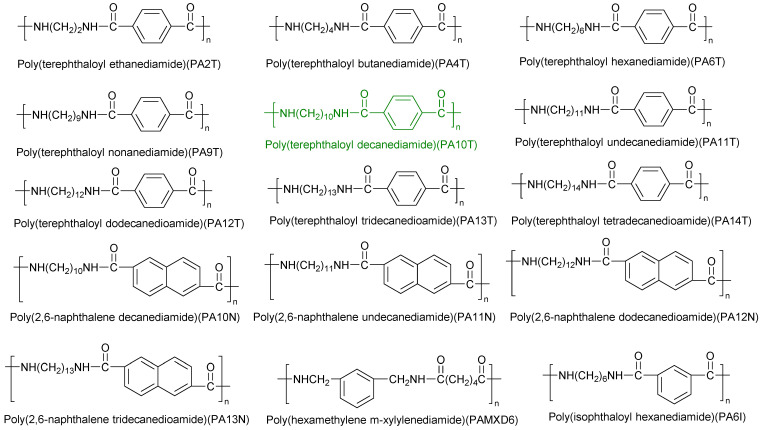
Common semi-aromatic polyamides. Commonly commercialized bio-based polyamides are highlighted in green.

Common semi-aromatic polyamides are shown in Figure 2. Semi-aromatic polyamides have both aromatic rings and aliphatic chains in their molecular main chains, combining the excellent performance of aromatic polyamides and the good molding processability of aliphatic polyamides. They generally have high temperature resistance, corrosion resistance, and low water absorption rate, and have been widely used in electronics, the automotive industry, equipment manufacturing, and other fields in recent years. The solid-phase condensation method and solution polymerization method are the main methods for preparing various semi-aromatic polyamide materials, while blending modification, copolymerization modification, and filling reinforcement modification are commonly used methods for modifying semi-aromatic polyamide materials [10].

**Figure 3:**
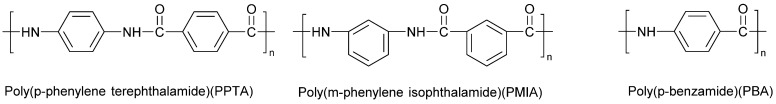
Common fully aromatic polyamides.

Common fully aromatic polyamides are shown in Figure 3. The synthesis of fully aromatic polyamides often uses low-temperature solution condensation or interfacial condensation. Poly(p-phenylene terephthalamide) fiber is made by dissolving the polymer obtained by condensation of p-phenylenediamine and terephthaloyl chloride in concentrated sulfuric acid, hydrofluoric acid, or chlorosulfonic acid to make a solution with liquid crystal properties, which is then made by dry–wet spinning. Poly(m-phenylene isophthalamide) fiber is made by dissolving the polymer obtained by condensation of m-phenylenediamine and isophthaloyl chloride in organic solvents such as tetrahydrofuran, dimethylacetamide, N-methylpyrrolidone, etc., and made by dry spinning or wet spinning. Fully aromatic polyamides are classified as high-performance materials due to their excellent mechanical strength and high heat resistance and are widely used in the military and other fields [11,12].

PA-6 and PA-66 account for about 90% of all polyamide products, and both are equally important [13]. Therefore, research on polyamides mainly focuses on these two polyamides, and this article also discusses these two polyamides.

**Figure 4:**
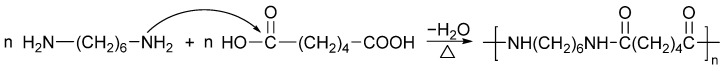
Possible mechanism for the synthesis of PA-66 by hexamethylenediamine and adipic acid [15].

**Figure 5:**
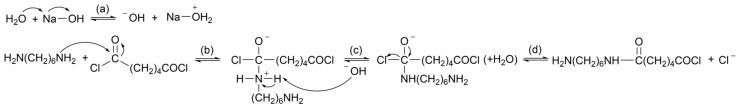
Possible mechanism for the synthesis of PA-66 dimer by hexamethylenediamine and adipoyl chloride [15]. (**a**) NaOH dissociates in water. (**b**) Nucleophilic attack. (**c**) OH^−^ catalyzes the reaction to proceed with interfacial condensation. (**d**) Removal of Cl^−^.

The polymerization process methods of PA-66 include solution–melt polymerization, high-temperature and high-pressure solution polymerization, interfacial polymerization, and solid-phase polymerization. At present, solution–melt polymerization is commonly used in industry to prepare PA-66. That is, first prepare a 30% hexamethylene diamine aqueous solution, and then add adipic acid with the same molar mass as hexamethylene diamine to prepare nylon 66 salt at 40–50 °C. The purpose of salting first is to remove impurities and ensure the molar ratio of hexamethylene diamine to adipic acid. The nylon 66 salt is made into a 50–60% aqueous solution, and a molecular weight regulator (generally acetic acid or adipic acid) is added. Pre-condensation at 230 °C and 1.7–1.8 MPa for 2 h. The reason for not directly melt condensing is that hexamethylene diamine is volatile, pressurization can suppress volatilization, and pre-condensation can generate PA-66 oligomers, then gradually depressurize, discharge water vapor, gradually increase the temperature, and increase the vacuum to reach a pressure of 0.1 MPa. The temperature must be 280 °C and the reaction must run for 45 min to obtain the product. This polymerization method can obtain high-molecular-weight polymers, it is easy to realize continuous production, and the product performance is stable. However, the high viscosity of the product, difficulty in discharging, high polymerization temperature, and high requirements for the gas tightness of the reactor and the polymerization process also limit the application of this polymerization method to a certain extent [14]. PA-66 has two main polymerization mechanisms. One is the amide condensation of adipic acid and hexamethylenediamine to form PA-66. The possible mechanism for the synthesis of PA-66 by hexamethylenediamine and adipic acid is shown in Figure 4. Hexamethylenediamine has strong nucleophilicity, and this reaction generally does not require catalysis. Another method is the interfacial amide condensation of aqueous hexamethylenediamine and oily adipoyl chloride to form PA-66. The possible mechanism for the synthesis of PA-66 dimer by hexamethylenediamine and adipoyl chloride is shown in Figure 5 [15].

**Figure 6:**
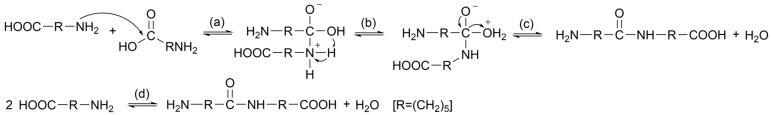
Possible mechanism for the synthesis of PA-6 dimer by 6-aminohexanoic acid [19]. (**a**) Nucleophilic addition. (**b**) Molecular rearrangement. (**c**) Dehydration. (**d**) Overall reaction.

**Figure 7:**
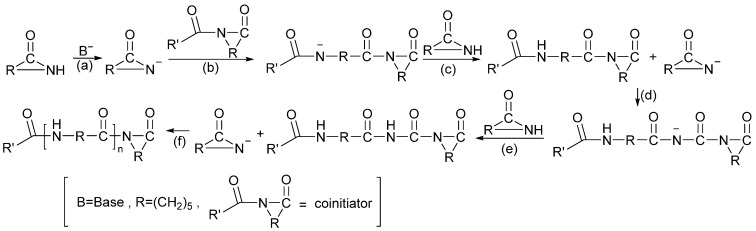
Possible reaction course of caprolactam anionic ring-opening polymerization [17]. (**a**) The base deprotonates to form an amide anion. (**b**) Nucleophilic attack on the acylimino carbonyl of the co-initiator (if present) or another amide carbonyl of the amide (if no co-initiator is used). (**c**) A proton is transferred from the unreacted amide to the amide enolate anion formed in the growing polymer backbone to regenerate the amide anion. (**d**) Increase in degree of polymerization. (**e**) Charge transfer. (**f**) Formation of polymers.

**Figure 8:**
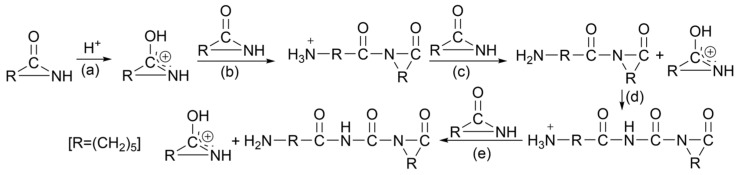
Possible reaction course of caprolactam cationic ring-opening polymerization [17]. (**a**) Polymerization begins with protonation of the amide, producing an electrophilic center. (**b**) Neutral amide attacks protonated amide, producing acylaminoamide cation as ammonium salt. (**c**) The ammonium cation protonates another amide monomer, thereby regenerating the cationic protonated amide and producing a neutral molecule with an amino end group. (**d**) Amide cation acylates neutral amine, promoting polymerization. (**e**) Charge transfer.

**Figure 9:**
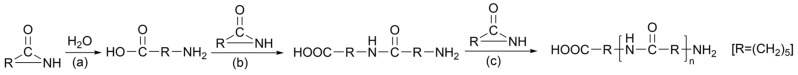
Possible reaction course of water-induced caprolactam ring-opening polymerization [18]. (**a**) Water initiation. (**b**) Increase in degree of polymerization. (**c**) Formation of polymers.

The polymerization process methods of PA-6 include hydrolysis polymerization, solid-phase polymerization, ionic polymerization, intercalation polymerization, etc. At present, hydrolysis polymerization is commonly used in industry. That is, caprolactam melts under a nitrogen gas flow and 0.5 MPa conditions, then it is heated to 267 °C, and water vapor is continuously added at 10 kg/h for 0.5 h. The output gas flow condenses in a container with water. The temperature and pressure are kept constant, and nitrogen is passed for 3 h. The reactor pressure is slowly reduced to terminate the polymerization, and finally, the molten polyamide is allowed to flow out of the reactor and cool [16]. PA-6 has two main polymerization mechanisms. One is the self-amidation condensation of 6-aminocaproic acid to form PA-6. The possible mechanism for the synthesis of PA-66 dimer from 6-aminohexanoic acid is shown in Figure 6. Another method is the ring-opening polymerization of ε-caprolactam (CPL) to form PA-6. Three types of kinetic mechanisms have been reported: hydrolysis (stepwise ring-opening), cationic, and anionic, respectively, using water, acid, and alkali as catalysts to initiate ring-opening. The possible reaction course of caprolactam anionic ring-opening polymerization is shown in Figure 7. The possible reaction course of caprolactam cationic ring-opening polymerization is shown in Figure 8. When water is used as an initiator, caprolactam is first converted to aminocaproic acid, which then attacks caprolactam to initiate the polymerization reaction. The possible reaction course of water-induced caprolactam ring-opening polymerization is shown in Figure 9 [17,18].

**Figure 10:**
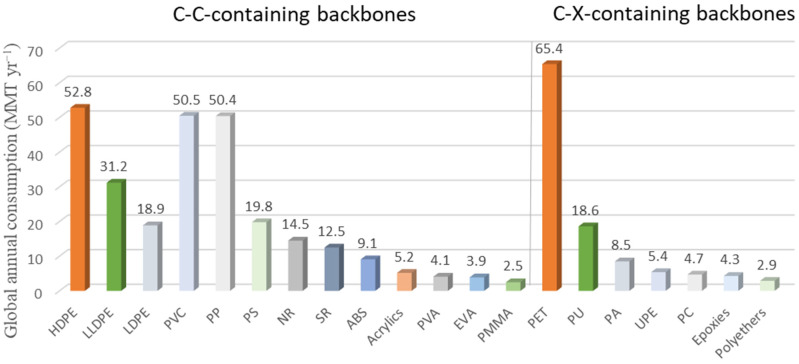
Annual global market size of commodity plastics in MMT yr^−1^ [21]. Polymers whose main chain is entirely C–C bonds: polyethylenes (including HDPE, LDPE, and linear LDPE (LLDPE)), PVC, polypropylene (PP), polystyrene (PS), natural rubber (NR), synthetic rubber (SR), acrylonitrile–butadiene–styrene (ABS), acrylics, poly(vinyl acrylate) (PVA), ethylene-vinyl acetate (EVA), and poly(methyl methacrylate) (PMMA) polymers whose main chain is entirely C–C bonds: PET, polyurethanes (PU), polyamide (PA), unsaturated polyesters (UPE), polycarbonate (PC), epoxies, and polyethers. All polymers included here have global annual market sizes that exceed 2.5 MMT yr^−1^.

PA has brought us many daily benefits in the fields of transportation, clothing, entertainment and health. But plastic waste in the environment also troubles people, especially the increasingly serious problem of microplastics nowadays [19]. According to statistics, the annual production of polymers (2016–2019) exceeded 2.5 million tons, and the annual production of PA was 8.5 million tons/year, ranking third in the production of polymers whose main chain is not entirely C–C bonds [20]. The annual output of various polymers is shown in Figure 10 [21]. In recent years, the development of PA has been very rapid, and its market size is expected to grow at a rate of 2.2%, reaching an annual output of 10.4 million tons by 2027 [22,23].

Microplastics generally refer to plastic particles or fragments with a size of less than 5 mm. Their sources include, but are not limited to, the following: production and processing of plastic products, decomposition of waste after use, and washing fibers. These microplastics may enter the environment through various channels such as rivers, oceans and soil, etc., and may have negative impacts on ecosystems and human health. According to existing research findings, polyamides account for up to 53.3% of microplastics in Vesijärvi Lake and Pikku Vesijärvi Pond near Lahti City in Finland [24]. Polyamides are also the most abundant plastics at five different drinking water sites with a share of 33%, and polyamides account for 17% of microplastics in Italian sewage treatment plants [25]. In addition, polyamide microplastics have also appeared in Black Sea sediments, beaches in San Rossore Massaciuccoli Natural Park (Pisa Italy), sludge from Chinese sewage treatment plants, river sediments and central Italian coasts [26,27,28,29,30]. Polyamide microplastics inside organisms have also been reported one after another. Polyamides are the most common polymers in pig lungs with a share of up to 46.11% [31], and microplastics account for up to 35.6% among ten fish species in the English Channel [32]. Polyamide microplastics have also appeared in the sediments and gastrointestinal tracts of Halibut roundworms and widely consumed Nile tilapia (*Oreochromis niloticus*) [33,34]. These microplastics not only harm marine animals but also change the food chain relationships in ecosystems, causing serious harm to ecosystems [35,36,37]. In addition, microplastic waste also poses a great threat to human health, including reproductive harm and obesity, as well as organ problems and delayed child development [38].

Various studies have shown that it is urgent to solve the environmental pollution caused by polyamide microplastics [24,25,26,27,28,29,30,31,32,33,34,35,36,37,38]. The main purpose of this review is to provide a comprehensive overview of the current status of polyamide recycling and degradation, including the reaction mechanisms and conditions in the energy recovery and recycling process and environmental degradation. A general conclusion has been drawn on the current situation, and suggestions have been made for the work to completely solve the polyamide pollution problem.

Valerian Hirschberg et al. [39] recently also published a paper on the polyamide recycling process and conditions, conducted an economic and technical analysis of different recycling methods of polyamides and compared their advantages and limitations. However, the current literature lacks a detailed summary of the synthesis, recovery, and degradation mechanisms of PA. This article summarizes the synthesis, recovery, and degradation mechanisms of polyamides, describes the industrial status of polyamide recovery, compares the products and technical bottlenecks of various current recovery and degradation methods, proposes possible solutions to existing PA pollution problems, and makes prospects for the future development trend of PA.

## 2. Energy Recovery and Recycling Process

The recovery of polyamides can be divided into monomer recovery (such as pyrolysis under alkali catalysis, acidic hydrolysis, alkaline hydrolysis, hydrothermal reaction, microwave-assisted hydrolysis, depolymerization in ionic liquids), reprocessing (such as mechanical recovery, solvent precipitation recovery), energy recovery (incineration), or conversion to other chemical raw materials (ammonolysis, alcoholysis, hydrogenation depolymerization). The specific means can be divided into energy recovery and pyrolysis, physical recovery and chemical recovery, considering that filling and landfilling is also a treatment method, so filling and landfilling are also included in this chapter.

**Figure 11:**
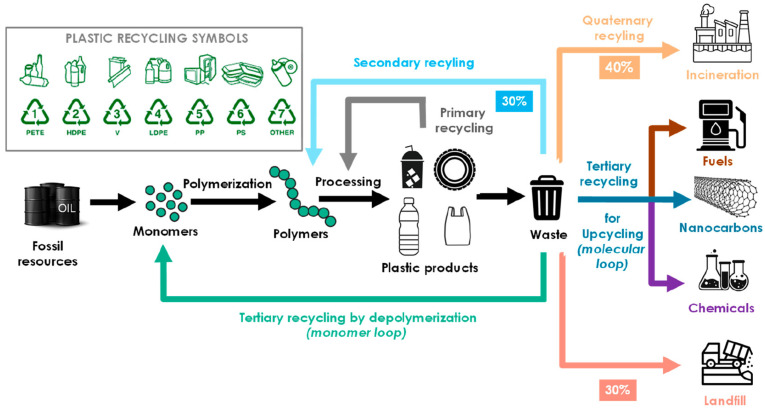
Life cycle of polymeric materials and main recycling technologies [34]. The number indicates the specific type of plastic and is used in the following manner: PET (1), HDPE (2), PVC (3), LDPE (4), PP (5), and PS (6). Code 7 is finally used to cover all other types of potentially recyclable plastics, e.g., polycarbonates (PC), polyurethanes (PU), polyamides (PA), and bioplastics such as polylactide (PLA), PMMA, or polyesters different from PET.

An effective way to treat waste such as polyamide fibers, resins and films is recycling and regeneration utilization [19]. Depending on different treatment methods, it can be divided into filling landfilling incineration physical or chemical recycling utilization. The life cycle of polymer materials and major recycling technologies is shown in Figure 11 [34,40]. For polymers whose main chain is entirely C–C bonds (such as polyethylene (PE), polypropylene (PP), polystyrene (PS), and polyvinyl chloride (PVC)), they are very resistant to degradation or hydrolysis, and their chemical recovery is also restricted [41]. Polymers, such as polyamides (PA), polyesters (i.e., PET and polycarbonate (PC)), and polyurethanes, are characterized by main chains that are not entirely C–C bonds and contain heteroatoms like O and N. Their hydrophilic amide or ester bonds make them susceptible to degradation in the presence of water. When disposed of in landfills, these polymers can persist for decades or even centuries [42]. This may be caused by three main reasons. First, the addition of additives (such as antioxidants and stabilizers) gives these polymer products antioxidant and anti-biodegradable characteristics. Second, biodegradation only begins when the molecular weight value reaches tens of thousands, and many commercial polymers have a molecular weight far greater than tens of thousands [43,44]. Lastly, the crystallization behavior of these polymers (such as PA and PET) can have a negative impact on biodegradation [45]. PET is the most recycled plastic in the world. Similar to PA, PET also has a certain degree of crystallinity, and both are typical condensation polymers. There are currently reports that enzymes can be used to depolymerize PET materials, and the progress of industrial composting research on PET is rapid [46,47,48,49,50,51]. Unfortunately, no enzyme has been found that can effectively degrade high-PA polymers [52,53,54,55]. From the perspective of molecular structure, the polarity of the C–O bond in the ester bond is greater than the C–N bond in the amide bond, and the cleavage activity of the C–O bond in the ester bond is greater than the C–N bond in the amide bond, which means that PA is more difficult to degrade than PET. The recycling methods of PET are also very similar to PA, physical recovery, acid–base hydrolysis, and alcoholysis are also applicable to PET. The knowledge and experience gained on PET may also be applied to solve PA problems [56]. 

The depolymerization of PA-6 was the earliest chemical recycling method implemented in industry. In 1993, BASF set up a PA-6 carpet recycling facility in Canada. This facility includes complex mechanical separation of PA-6 fibers (including dry and wet process) and subsequent depolymerization process. There are mainly two types of depolymerization processes: one is depolymerization under high temperature and reduced pressure in the presence of acid or alkali organic solvent (acidic hydrolysis or alkaline hydrolysis mechanism); the other is depolymerization under conditions without acid or alkali in the presence of water at 270–350 °C (hydrothermal reaction mechanism) [57,58,59,60]. Subsequently, DuPont, Rhodia Performance Fibres, Polyamid2000, DSM, and AlliedSignal also set up PA-6 and PA-66 recycling production lines [61,62,63,64,65]. 

PA accounts for about 10% of marine debris, and according to the World Animal Protection Association, over 600,000 tons of fishing gear, including nylon nets discarded by fishermen, are dumped into the ocean each year. In the search for effective industrial solutions to recycle this synthetic fiber, Aquafil has made outstanding contributions. In 2007, they began developing a machine that could stir most polyamides, producing new lines for reuse. In 2012, the Econyl^®^ product was launched, marking the start of a “closed cycle” that can recycle products made from PA-6 and regenerate them into more sustainable raw materials. Recycled waste such as fishing nets is first sent to a pre-treatment facility, where they are sorted and crushed into small enough pieces. Then, the shredded material is transferred to a regeneration factory, where they are put into a large chemical reactor. Through the process of depolymerization and repolymerization, the components of the material are broken down and PA-6 is regenerated, which is then processed into yarn and can finally be made into premium bags [66,67].

Today, the mechanical recycling method is technically mature, economically beneficial, and easy to operate. Most companies on the market use this method, which is currently the mainstream method for recycling waste polyamides. However, the performance degradation caused by mechanical recycling is unavoidable, and if the waste polyamide is contaminated, it will also lead to the inability to recycle. The requirements for chemical recycling technology are high, and most large-scale recycling manufacturers are in some developed countries [68,69]. Zimmer (Germany) has established a 20,000 t/a PA-6 acid depolymerization recycling production line, BASF (Germany) has established a 20,000 t/a PA-6 acid depolymerization and alkali depolymerization, 24,000 t/a PA-66 alkali depolymerization and 590 t/a PA-6 acid depolymerization recycling production line, Rhône-Poulenc Société Anonyme (France) has established a 50,000 t/a PA-66 alkali depolymerization recycling production line, and Dupont (USA) has established a 230 t/a PA-66 methanol alcoholysis depolymerization recycling production line [70,71,72].

### 2.1. Filling and Landfilling

Of the plastics produced from 1950 to 2015, only 9.5% were recycled, 12.5% were incinerated, and as much as 78% were buried in landfills [73]. When polyamides are buried in landfills, they will exist for at least several decades, or even several centuries [42]. This may be the result of the combined effects of additives, molecular weight, and crystallization [43,44]. Moreover, due to the nature of polyamide waste itself it easily decomposes to produce harmful gases and liquids causing environmental pollution. Therefore, landfilling treatment for polyamide waste is not mainstream or advocated for now [74].

Research on filling polyamides includes filling alumina fibers with PA-66 filling multivalent alcohols with PA-11 and PA-12 and layered silicate filling waste PA-12 nanocomposites, etc. [75,76]. However, in terms of the polyamide waste produced every year, this consumption is nothing more than a drop in the bucket, and filling cannot fundamentally solve the pollution problem of polyamides.

### 2.2. Energy Recovery and Pyrolysis

The main way for polyamides to be converted into energy for reuse is incineration, and the main way to convert them into polymer monomers for recycling is pyrolysis.

Incineration can recover energy through the gases and oils produced during the process. The incineration of polyamides only produces 30.2 MJ kg^−1^ of energy [77]. However, the production of polyamides is energy-intensive, and it takes 163 MJ Kg^−1^ of polymer to produce PA-66 [78]. It can be seen that the efficiency of incineration of polyamides is very low. During the combustion process, it may produce polluting toxic gases such as CO and NOx, etc., and the cost of treating these harmful wastes has increased [79], so incineration is not the best choice for recycling methods.

**Figure 12:**
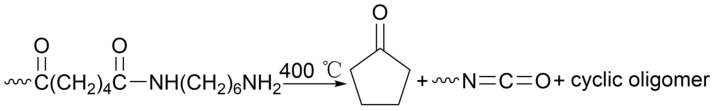
PA-66 pyrolysis reaction [80].

The key to producing pyrolysis fuel is how to avoid material cross-linking. By studying the thermal degradation of PA-6 at different heating rates, it was found that the yield of pyrolysis oil is highly related to temperature [79]. Less pyrolysis oil and more gas are produced at lower temperatures. The gas mainly consists of low-molecular-weight hydrocarbons, H_2_, CO and CO_2_. The main product of the slow pyrolysis of PA-66 at 400 °C is cyclopentanone. In addition, products with terminal nitrile and isocyanate groups will be formed, as shown in Figure 12. Cross-linking reactions will also occur between terminal isocyanates [80]. The use of these methods is limited by energy consumption and the characteristics of the final product.

### 2.3. Physical Recycling

#### 2.3.1. Mechanical Recycling

**Figure 13:**
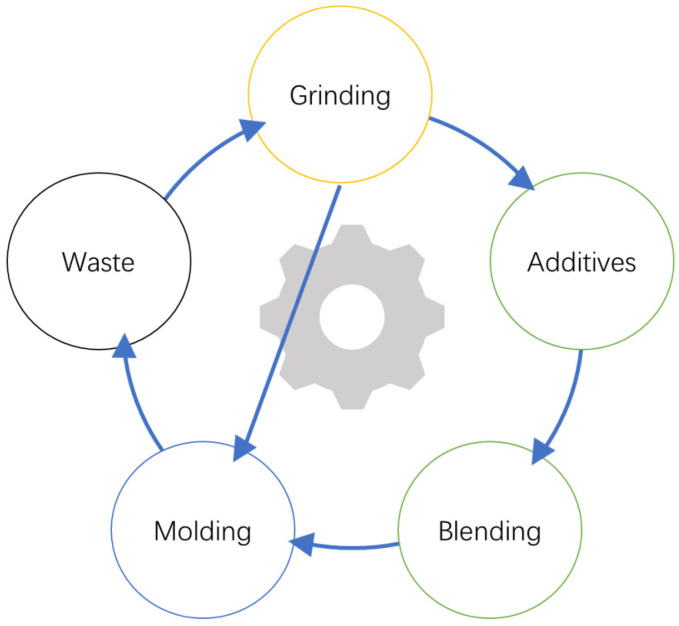
Overview of the mechanical recovery steps.

Mechanical recycling (secondary recycling) refers to the transformation of waste plastics through mechanical methods, generally completed by grinding machines, shredders, extruders, etc. Mechanical recycling has low processing costs, produces less residue, and requires less energy but produces pollutants. The general steps of mechanical recycling are shown in Figure 13. After grinding polyamide waste, additives are added to blend or directly mold [78].

Early research on mechanical recycling was about ABS, PA, PC, and PMMA [81]. First, a knife mill is used to grind dry waste materials after extrusion in a twin-screw extruder, and finally, injection molding is used. Bernasconi et al. [82] studied glass fiber with a mass fraction of 35% PA-66 and found that when the content of recycled materials accounts for 50% of the material, the performance loss is less than 5%, but when the content of recycled materials accounts for 100% of the material, the performance loss is 14%. Through mechanical recycling of carbon fiber reinforced with a mass fraction of 30% PA-66, it was found that mechanical recycling can only degrade original samples to a limited extent, and aging causes significant performance loss [83]. Table 1 shows the literature related to mechanical recycling with polymer types, reinforcing materials, and processing methods [78].

#### 2.3.2. Solvent Precipitation Recovery

Solvent precipitation recovery refers to the method of dissolving a material into a solvent or combination thereof for easier separation and disposal. Using different solubility of different components in solvents, pure materials can be obtained, which may be highly effective for blends and composites. However, due to crystallization and other reasons, the solubility of PA is quite poor. Common solvents that can dissolve PA include hexafluoroisopropanol (HFIP), hexamethylphosphoramide (HMPA), ionic liquids, formic acid (FA), phenol, trifluoroethanol, α-cyanoalcohol, etc. [98]. Hexafluoroisopropanol is widely used in gel permeation chromatography analysis of PA, while hexamethylphosphoramide can serve as a co-solvent for synthesizing aromatic polyamides. Additionally, ionic liquids can be employed for depolymerizing PA. The poor solubility of PA significantly limits the widespread adoption of solvent precipitation recovery methods.

Studies have shown that PA (powder) can be recovered by reverse solvent precipitation using supercritical carbon dioxide at 40 °C under a pressure of 84–125 bar [99]. PA will also selectively dissolve in formic acid aqueous solution [100]. It can also dissolve PA-6 in DMSO at 110–130 °C, using methyl ethyl ketone as an anti-solvent to recover two polymers [101]. Although the above physical recycling through dissolution or precipitation has not significantly changed the physical and chemical properties of PA, these methods have strict reaction conditions or use chemical reagents that cause pollution, so it is necessary to find more energy-saving and environmentally friendly recycling methods.

### 2.4. Chemical Recycling

Chemical recycling of PA is one of the focuses of research. The currently reported chemical recycling methods for PA include alkaline hydrolysis, hydrothermal reaction, microwave-assisted hydrolysis, acidic hydrolysis, ammonolysis, alcoholysis, hydrogenolysis polymerization, depolymerization reaction in ionic liquids, etc. [78].

Although polyamides are difficult to hydrolyze under normal circumstances, because polyamides contain amino and carbonyl groups, they can easily form hydrogen bonds with water molecules, so the resulting materials can easily absorb water when used. The most commonly used PA-6 and PA-66 can absorb up to 10% of water from humid air and can absorb 2% to 4% of water in a general humidity environment [19]. When water is combined with other factors, the hydrolysis rate of polyamides can be significantly increased. When there is acid or alkali in the water, it is acid hydrolysis or alkaline hydrolysis. Polyamides can also accelerate the reaction when heated in water.

#### 2.4.1. Alkaline Hydrolysis

**Figure 14:**
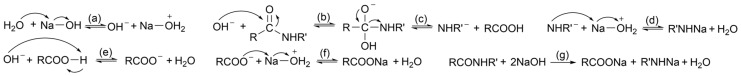
Possible mechanism of amide alkaline hydrolysis [105,106]. (**a**) NaOH ionizes in water. (**b**) Nucleophilic addition. (**c**) Nucleophilic elimination. (**d**) Nucleophilic substitution. (**e**) Acid-base neutralization. (**f**) Nucleophilic substitution. (**g**) Overall reaction.

Polyamide materials have strong alkali resistance. Manas Ranjan Puha et al. analyzed polyamide-based membranes under pH 13 conditions. X-ray photoelectron spectroscopy (XPS) analysis of the product groups showed that the amide bond was not decomposed [102]. However, polyamides will also degrade under stronger alkaline conditions. This can be attributed to the degradation of incompletely crystallized segments in polyamide fibers or molecules on the one hand and microcrystalline degradation on the other hand [103]. In terms of physical performance, it is manifested as a decrease in breaking strength and breaking energy as the alkali concentration increases [104]. The possible mechanism of amide alkaline hydrolysis is shown in Figure 14 (unless otherwise specified, R and R’ appear below to represent alkyl or aryl groups).

Although alkaline hydrolysis can decompose polyamide materials into small molecules that can be degraded in a short period of time in natural environments, the required alkali solution concentration is too high, which not only greatly increases the cost of polyamide degradation but also causes environmental pollution during the preparation of alkali drugs. This obviously does not meet people’s original intentions, so we need to find a more economical and environmentally friendly plan.

#### 2.4.2. Acidic Hydrolysis

**Figure 15:**
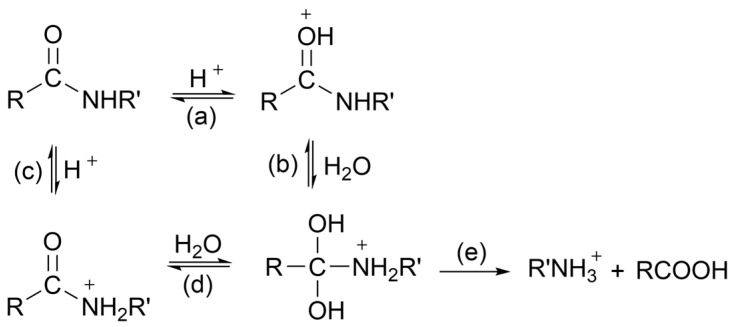
Possible pathways for amide acid hydrolysis [105]. (**a**) Nucleophilic attack. (**b**) Nucleophilic addition. (**c**) Nucleophilic attack. (**d**) Nucleophilic addition. (**e**) Nucleophilic elimination.

Many studies have shown that acid concentration is critical to polyamide hydrolysis. Some polyamide materials can be used under less extreme acidic conditions. Through research on acidic conditions for polyamides, it was found that PA-11 will only undergo significant degradation at 90 °C and pH < 2 [107,108,109]. Generally speaking, the greater the acid concentration, the greater the rate of polyamide hydrolysis [110,111]. But some studies have also found that under the same pH value, different types of acids will also affect the rate of polyamide hydrolysis. The reason for this result may be due to different similarities between different acids and polyamides and different solubility for polyamides [112]. The mechanism of acid catalysis of amide is not fully understood, but it is generally believed that the amide is protonated on the O first, although there are also theories that it is protonated first on the N. There may be two possible pathways for amide acid hydrolysis, as shown in Figure 15 [105].

#### 2.4.3. Hydrothermal Reaction

The research on the hydrothermal reaction of polyamides originated from the problems encountered in the synthesis of PA-6. Generally, PA-6 is processed and prepared into plastics or fibers in a molten state. However, due to reasons such as the high crystallinity of PA-6, PA-6 only dissolves in a few strong polar solvents. High cost and environmental pollution make PA-6 unsuitable for processing in solution. Water is an environmentally friendly solvent. Although PA-6 is insoluble in water at room temperature, it can dissolve in superheated water under pressure [113,114,115].

**Figure 16:**
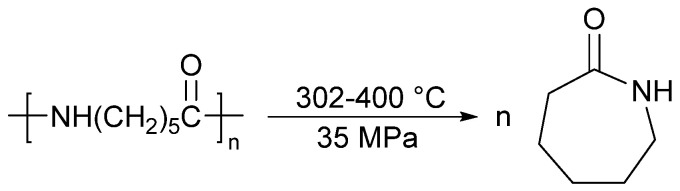
PA-6 hydrothermal reaction equation [114].

Research on the hydrothermal reaction of PA-6 emerged as required. When the temperature is above 160 °C, PA-6 will dissolve in water under pressure [116]. During the dissolution process, the amide group will undergo hydrolysis, and the use of hydrothermal reaction to recover PA-6 has also been proposed [114,117,118,119,120,121,122,123,124]. When the temperature reaches 302–400 °C and the pressure reaches 35 MPa, PA-6 can also degrade into α-caprolactam. The total reaction formula for the PA-6 hydrothermal reaction is shown in Figure 16 [114]. The hydrothermal reaction does not use environmentally polluting reagents, but the high temperature and high pressure reaction conditions also make its cost expensive. If the energy consumption of the hydrothermal reaction can be significantly reduced, the environmental pollution problem of PA may be effectively solved.

#### 2.4.4. Pyrolysis under Alkali Catalysis

**Figure 17:**
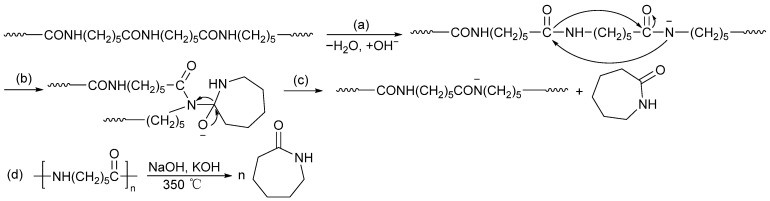
Anionic degradation of PA-6 in the presence of NaOH/KOH bases [69]. (**a**) Deprotonation. (**b**) Nucleophilic attack. (**c**) Intramolecular cyclization. (**d**) Overall reaction.

The idea of recycling monomers from polyamides is to heat and break the C–N bond in the amide bond [125]. At present, it is only applicable to PA-6. The polymerization method of PA-6 is ring-opening polymerization without producing water, while water is produced during the polymerization process of diamine-dicarboxylic acid series polyamides, so this method is most likely only suitable for lactam series polyamides. The current ideal conditions for pyrolyzing PA-6 are using NaOH and KOH as catalysts, and the monomer yield obtained at 350 °C is 98.4%; its reaction formula is shown in Figure 17. The mechanism of alkaline-catalyzed PA-6 depolymerization under high temperatures may be anionic degradation. First, the amide groups are deprotonated, followed by intramolecular cyclization to form lactam units [71,126]. The pyrolysis method has achieved good results in recycling PA-6 monomers, but using NaOH and KOH as catalysts and conditions at 350 °C greatly increases the recycling cost.

#### 2.4.5. Microwave-Assisted Hydrolysis

Microwaves generally refer to electromagnetic waves with a frequency range of 300 MHz~300 GHz. Under this frequency of electromagnetic waves, the orientation of polar molecules will change with the frequency of the alternating electromagnetic field, and the movement and friction between molecules will generate heat [127].

Urška Češarek et al. [128] found in their study of the chemical recycling of aliphatic polyamides that when HCl was used as an acid catalyst with external microwave radiation at 200 °C and a 1.25 HCl/amide molar ratio, PA-66 could be completely converted into constituent monomers in 10 min. Subsequently, Eva Bäckström et al. [129] found that polyamide-6 (PA-6) and polyamide-66 (PA-66) would be selectively hydrolyzed by microwave-assisted hydrolysis of industrial multi-component polyamide-6 (PA-6)/polyamide-66 (PA-66)/polypropylene (PP) carpets, which may provide new ideas for the separation and degradation of composite materials. 

By analyzing the products after the hydrolysis of PA-6 and PA-66 by MALDI-TOF, it was found that PA-6 decomposes into caprolactam and low-molecular-weight compounds with NH_2_ or COOH termini, while PA-66 decomposes into three types of low-molecular-weight compounds with termini, namely low-molecular-weight compounds with NH_2_/COOH termini, low-molecular-weight compounds with NH_2_/NH_2_ termini, and low-molecular-weight compounds with COOH/COOH termini [130].

Microwave-assisted hydrolysis of polyamides has also achieved good results in the recycling process of polyamides and may have the ability to separate polyamide composite materials. The disadvantage is that acid is still needed as a catalyst, and energy consumption by microwaves is also an unavoidable problem.

#### 2.4.6. Ammonolysis

The earliest research on ammonolysis depolymerization of polyamides was two patents published by DuPont between 1994 and 1995. The reaction used phosphate or homogeneous Lewis acid (ScCl_3_ or TiCl_4_) as a catalyst. The reaction temperature and pressure were 320 °C and 138 bar, respectively. The monomer yields of PA-6 and PA-66 were 88% and 61%, respectively [131,132].

**Figure 18:**
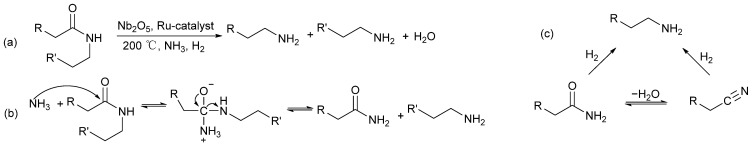
(**a**) Amide ammonolysis reaction. (**b**) Possible mechanism of amide aminolysis. (**c**) Reactions involving hydrogen in PA ammonolysis. Ref. [133].

Subsequently, research on the ammonolysis of polyamides focused on reducing the reaction conditions for the ammonolysis of polyamides. Robin Coeck et al. recently proposed a multiphase catalytic system based on Nb_2_O_5_. The reaction temperature was reduced to 200 °C. The reaction formula is shown in Figure 18a. The possible mechanism of amide aminolysis is shown in Figure 18b. This high-molecular-weight chain containing terminal amide groups can undergo reactions as shown in Figure 18c. Under the action of hydrogen gas, terminal amide groups can be converted into terminal amino groups. In addition, high-molecular-weight chains containing terminal amide groups will also dehydrate to form high-molecular-weight chains containing terminal cyano groups [133].

Ammonolysis has achieved undeniable achievements at the mechanistic level. It provides us with a brand new method for degrading polyamides. However, in terms of its reaction conditions, high reaction costs will inevitably limit its development.

#### 2.4.7. Alcoholysis

**Figure 19:**
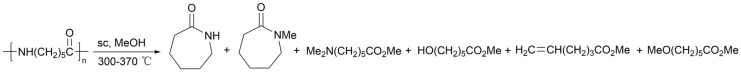
PA-6 alcohololysis reaction [134].

Akio Kamimura’s team has conducted in-depth research on the alcoholysis of polyamides. In 2011, this team directly converted polyamides into hydroxyalkanoic acid derivatives. The total reaction formula is shown in Figure 19. Supercritical methanol treatment of PA-6 produces six types of products: caprolactam, N-methylcaprolactam, 6-(N,N-dimethylamino)hexanoic acid methyl ester, 6-hydroxyhexanoic acid methyl ester, 5-hexenoic acid methyl ester, and 6-methoxyhexanoic acid methyl ester. The proportions of each product vary depending on the reaction temperature. By studying the relative amounts of each product during the reaction process, caprolactam is found to be the primary intermediate. N-methylcaprolactam and 6-(N,N-dimethylamino)hexanoic acid methyl ester increase with a decrease in caprolactam content. 6-hydroxyhexanoic acid methyl ester and 5-hexenoic acid methyl ester are the final products, while the content of 6-methoxyhexanoic acid methyl ester is close to 0% [134].

**Figure 20:**
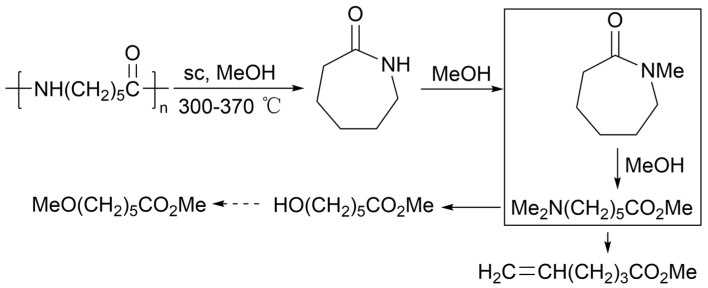
Possible reaction course of PA-6 alcoholysis [134].

The possible reaction course of PA-6 alcoholysis is shown in Figure 20. PA-6 first opens the chain to form caprolactam, and then caprolactam reacts with methanol to produce N-methylcaprolactam or 6(N,N-dimethylamino)caproic acid methyl ester. Among them, N-methylcaprolactam can react with methanol to open the ring to produce 6(N,N-dimethylamino)caproic acid methyl ester. Upon further heating, the N,N-dimethylamino group in 6-(N,N-dimethylamino) is eliminated, resulting in the formation of either 6-hydroxyhexanoic acid methyl ester or 5-hexenoic acid methyl ester. 6-hydroxyhexanoic acid methyl ester can react to produce 6-methoxyhexanoic acid methyl ester, but this process is slow. It is important to note that in the later stages of the reaction, these reactions are not complete, and all six products coexist simultaneously [134].

**Figure 21:**
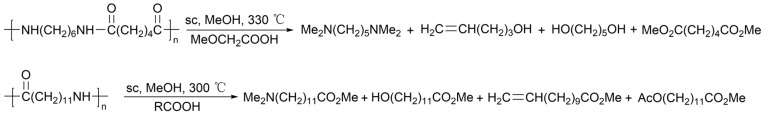
PA-66 and PA-12 alcoholysis reaction [135].

In 2014, the team also studied the alcoholysis reaction of PA-66 and PA-12. The reaction formula is shown in Figure 21 [135]. PA-66 is treated with methoxyacetic acid and supercritical methanol at 330 °C to produce N,N,N,N’-tetramethyl-1,5-pentanediamine, 1,6-hexanediol, 5-hexen-1-ol and adipic acid dimethyl ester [136]. PA-12 is treated with carboxylic acid and supercritical methanol at 300 °C to produce dimethylamino dodecanoic acid, 12-hydroxydodecanoic acid, 11-dodecenoic acid, and esters. Alcoholysis can degrade PA into monomer derivatives, but the harsh reaction conditions and the diversity of products limit the large-scale application of alcoholysis.

#### 2.4.8. Hydrogen Dissolving Polymerization

**Figure 22:**
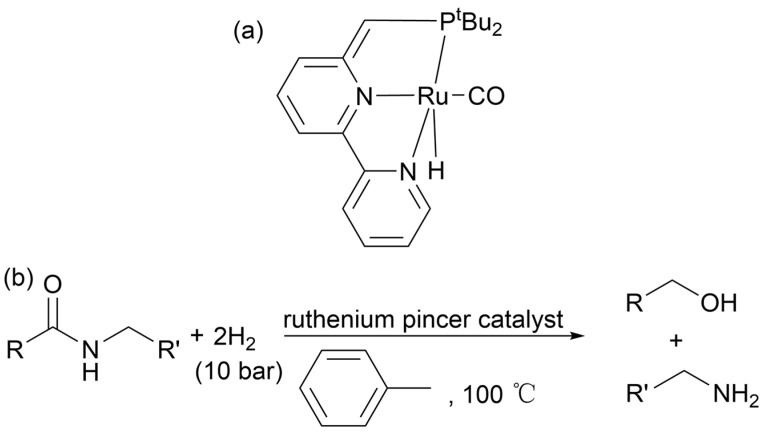
(**a**) Ruthenium clamp catalyst structure formula. (**b**) PA hydrogen dissolving polymerization reaction. Ref. [156].

Catalytic hydrogenation is an atom-economical, green, and sustainable conversion pathway that does not produce stoichiometric waste and hydrogen can be prepared from renewable resources [137]. The literature reports that ruthenium chelating catalysts (structure shown in Figure 22a) catalyze the hydrogenation of nylon C–N bonds to break and form alcohols and amines. The reaction formula is shown in Figure 22b [138]. In addition, there are also reports of homogeneous catalysts based on ruthenium [139,140,141,142,143,144,145,146,147,148], iron [149,150,151,152], molybdenum [153], and other homogeneous catalysts for hydrogenating amides to form alcohols and amines [154,155]. However, most conventional nylon materials such as PA-6, PA-66, or PA-12 have not been reported for catalytic hydrogenation. The reason may be that these polyamides have good solvent resistance. The depolymerization of PA during the ruthenium-catalyzed hydrogenation process has also been extensively studied [156].

#### 2.4.9. Depolymerization in Ionic Liquids

**Figure 23:**
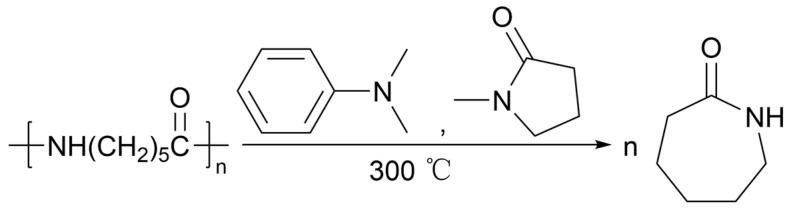
PA-6 reaction in ionic liquids [157].

Due to the non-volatility and stability of ionic liquids at high temperatures, some scholars have proposed using the characteristics of ionic liquids to depolymerize polyamides and speculate that the counter anions in ionic liquids play an important role in depolymerization. It was found that with DMAP (4-dimethylaminopyridine) as a catalyst and N-methylpyrrolidone as a solvent, the yield of caprolactam could reach 86% after reacting for 6 h at 300 °C. The reaction formula is shown in Figure 23 [157]. The advantage of ionic liquids is that they can be reused at least five times without significant decomposition and do not require high-pressure equipment. However, the reaction temperature and product separation also limit the widespread use of ionic liquids.

## 3. Environmental Degradation

Chemical or physical methods of human intervention have insurmountable difficulties in energy conservation, economy, pollution in the production process of chemical reagents, etc., so environmental degradation of polyamides may be a more appropriate choice.

### 3.1. Weather Degradation

Weather degradation generally refers to widespread degradation in nature, such as decomposition under the action of water, oxygen, acid–base salt electrolytes, sunlight, ozone, etc. Research on PA focuses on thermal oxidation degradation and degradation in natural environments [158,159].

#### 3.1.1. Thermal Oxidative Degradation

Many PA composite materials are exposed to heat for a long time and exposed to air. Research on PA thermal oxidation has emerged as required. PA10T/GF/FR composite material will undergo micro-crosslinking and aging at 200 °C under the PA molecular chain [160]. Poly(p-phenylene terephthalamide) (PA10T)/glass fiber (GF) composite material will significantly degrade its performance at 160 °C [161]. In addition, through research on PA-6/epoxy resin nanocomposite materials [162], molded sepiolite/PA-66 nanocomposite materials [163], PA-6/LGF composite materials [164], PA-6/graphene nanocomposite materials [162], short fiber reinforced polyamide composite materials [165], PA-6/halloysite nanotube composite materials [166], long glass fiber reinforced PA10T composite material [167], and PA-6/oxidized graphene nanocomposite material found that thermal oxidation degradation will significantly degrade the performance of the material [168].

By detecting the degradation products of PA-66 thermal oxidation aging with isotope labeling and gas chromatography-mass spectrometry, it was found that the mechanism of thermal oxidation degradation of PA-66 is very complicated. By analyzing the types of products obtained, substances such as 1-pentene, 2-pentanone, pyridine, 2-hexanone, benzene, cyclopentene, tetrahydropyran, butene, 2-butanone, THF, butane, acetone, methyl acetate, cyclopentanone, etc., were found. Gregory Von White II et al. also described the mechanism of each substance reaction in detail [169,170].

Although thermal oxidation degradation can degrade PA to a certain extent, due to limited degradation degree and too many products after degradation, which are not conducive to separation and recovery, it cannot effectively solve PA’s environmental pollution problem.

#### 3.1.2. Other Natural Environmental Degradations

Similar to thermal oxidation degradation, PA-12 will also cause significant performance degradation when exposed to ultraviolet light for a long time [171]. There are also reports that some polyamides can be degraded by certain microorganisms [172,173,174,175], but these conditions are very rare in nature, so it is difficult to use this as a basis for large-scale treatment of polyamide waste. Fortunately, PA-4 can be degraded in activated sludge, separating degrading bacteria from soil. Naoko Yamano et al. implanted PA-4 into rats, and after only 8.5 months, PA-4 was completely degraded in the rats [52,53,54,55]. In addition, they also tested the degree of degradation of PA-4 in seawater only after six weeks PA-4 was degraded by 70%. Unfortunately, there are no reports on the degradation of dominant PA-6 and PA-66 in common natural environments [176,177].

### 3.2. Enzymatic Hydrolysis

Since most polyamide products cannot be degraded in the natural environment, enzymatic degradation of polyamides has become a research hotspot in the industry. Currently reported enzymes that can degrade low-molecular-weight polyamides mainly include proteases, cutinases, and amidase. These enzymes can only act on the surface of polyamides. Low-molecular-weight polyamide hydrolase has not directly measured the depolymerization activity of polyamides. Surface hydrolysis of low-molecular-weight polyamides can increase the hydrophilicity of polyamides, so changes in hydrophilicity can indirectly prove the depolymerization of polyamides [34]. Another type of enzyme promotes the oxidative decomposition of polyamides to degrade polyamides [178,179,180,181].

PA fibers are hydrophobic, so many biocatalytic methods have been developed to change the hydrophobicity of polyamides. Proteases, cutinases, and amide hydrolases all achieve the goal of increasing hydrophilicity by forming hydrophilic groups (such as amino and carboxyl groups) on the surface of polyamides [178,179,182].

#### 3.2.1. Proteases

Protease is a general term for a class of enzymes that hydrolyze protein peptide chains. It is divided into endopeptidase and exopeptidase according to the way it degrades peptides. The former can cut large-molecular-weight peptide chains from the middle to form smaller-molecular-weight peptides and polypeptides; the latter can be divided into carboxypeptidase and aminopeptidase, which, respectively, hydrolyze peptide chains from the free carboxyl terminal or free amino terminal of peptides to generate amino acids. Proteases that have been reported to slightly hydrolyze PA-66 include papain, trypsin, and α-chymotrypsin [183]. There are also studies using bromelain to hydrolyze polyamide fibers, producing amino acids [184]. Aspartic protease, metalloprotease, and cysteine protease are also used for PA textile modification. Papain is a protease discovered by Genencor (including bromelain, Purafect OX 4000 E, Protease GC 106, Protex Multiplus L four proteases). Protease M was discovered by Amano. Corolase N was discovered by AB Enzyme. Flavor protease 500 L was discovered by Novozymes [185]. Alcalase 2.4 L protease, discovered by Novozymes, can also be used to modify the surface of PA-66 fibers [186].

#### 3.2.2. Cutinases

Cutinase is an α/β hydrolase belonging to serine esterase. It can degrade keratin and produce a large number of fatty acid monomers. Cutinase can catalyze the hydrolysis of insoluble plant keratin ester bonds as well as other long-chain and short-chain fatty acid esters, emulsified triglycerides and soluble synthetic esters. Cutinases that have been reported to degrade polyamides include GCI 2002/1410 cutinases discovered by Genencor and FsC cutinases from Fusarium oxysporum [187,188,189]. FsC cutinases are active against adipic acid bis-hexylamide, but have a 4 times lower activity than proteases from Bacillus due to its 3 times lower adsorption [187].

#### 3.2.3. Amidase

Studies have shown that five enzymes capable of catalyzing PA-6 hydrolysis have been identified in *Arthrobacter* sp. [190,191,192,193].

1. NylA 6-aminocaproate-cyclic-dimer (Acd) hydrolase hydrolyzes amide bonds at an optimal pH and temperature of 7.4 and 34 °C, respectively, producing 6-aminocaproate-linear-dimer (Ald). The X-ray structure of Acd hydrolase is in free form and is complexed with Acd. Studies have shown that the catalytic function of Acd hydrolase originates from the catalytic center composed of the S174/S150/K72 triad [194].

2. NylB 6-aminocaproate-linear dimer (Ald) hydrolase hydrolyzes amide bonds at an optimal pH and temperature of 9.0 and 40 °C, respectively and has activity against trimers to twenty-mers, producing 6-aminocaproic acid (Ahx). Ald hydrolase is more active against Ahx-Aoc (6-aminohexanoyl-8-aminooctanoic acid ester) and Ahx-Ani (6-aminohexanoyl-aniline) than against Ald but has almost no activity against Ahx-Aoc (4-aminobutyryl-6-aminohexanoic acid ester) or Ahx-Aoc (8-aminooctanoyl-6-aminohexanoic acid ester) [195]. Ald hydrolase has no activity against Acd or cyclic low-molecular-weight polyamides.

**Figure 24:**
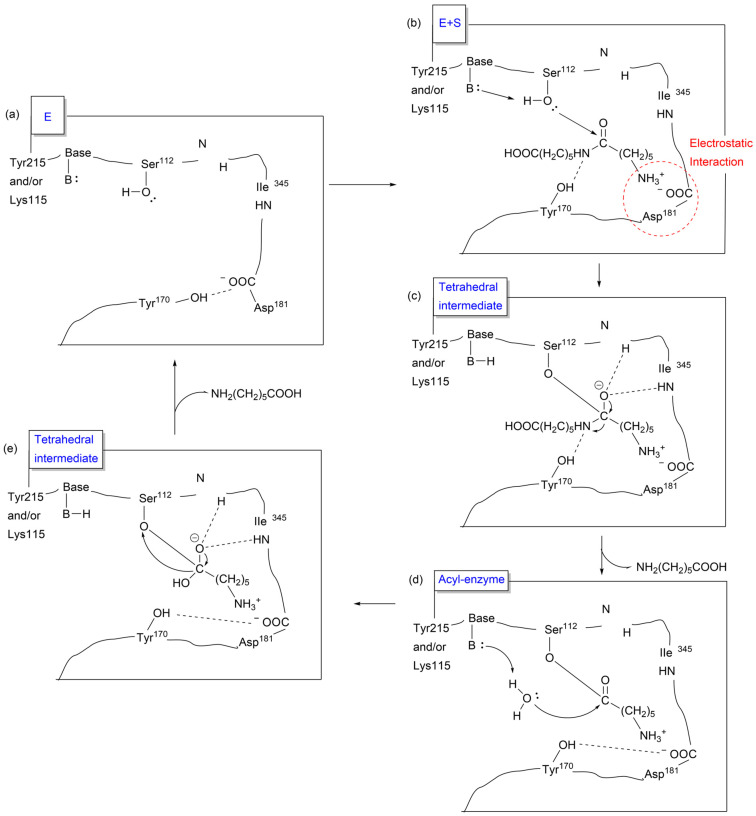
Proposed catalytic mechanism of NylB 6-aminocaproate-linear dimer (Ald) along the following steps: (**a**) free enzyme in open form, (**b**) enzyme + substrate, (**c**) tetrahedral intermediate in closed form (**d**) acyl-enzyme, and (**e**) tetrahedral intermediate in open form. Tyr215, Lys115, Ser112, IIe345, and Asp181 are the actual possible reaction sites in the enzyme. Ref. [53].

The catalytic mechanism of the NylB 6-aminocaproate-linear dimer (Ald) has been proposed to involve the following steps: (a) interaction between the catalytic center and N-ter of Ald; (b) induction of a conformational change by Ald in the enzyme from open form to closed form; (c) a nucleophilic attack on Ald by S112 to form a tetrahedral intermediate; (d) then, an acyl enzyme is formed with an open form enzyme; and (e) subsequently, the enzyme is deacylated by a water molecule and the free enzyme is regenerated by forming a tetrahedral intermediate. The proposed catalytic mechanism of NylB 6-aminocaproate-linear dimer (Ald) is shown in Figure 24 [190].

3. NylB’ has 88% amino acid sequence identity with Ald hydrolase, with only 46 different amino acids, but NylB’ has 200 times lower activity than Ald hydrolase.

4. NylC Ahx-oligomer endonuclease hydrolyzes amide bonds at an optimal pH and temperature of 7.0 and 42 °C, respectively, and can hydrolyze cyclic and linear oligomers [194].

5. Similar to NylC amide hydrolase, in addition, when studying the hydrophobicity modification of polyamide fabrics with asparaginase (AA) and endopeptidase (trypsin, TR), it was also found that AA hydrolysis caused amide bond cleavage and a large amount of amino acids were released into the reaction mixture. However, compared with AA hydrolysis, TR hydrolysis showed relatively low activity. Nocardia polyamide hydrolase can also partially hydrolyze amide bonds to produce surfaces with amines and carboxylic acids [196,197].

Using high-performance liquid chromatography (HPLC) to analyze the hydrolysis products of Nocardia polyamide hydrolase on PA, the monomer hydrolysis product adipic acid was found. The structural characteristics of polyamide hydrolase have not yet been elucidated. The active site of keratinase is exposed on the outside, while simulation by SWISS-MODEL found that the catalytic site of Nocardia polyamide hydrolase is located inside the enzyme, and further research into this aspect is needed [198].

The development of polyesterase precedes that of polyamide hydrolase, so some people have proposed to convert the reaction specificity of polyesterase into amide bond hydrolysis through enzyme engineering. It has been proposed that a recombinant water network obtained through enzyme design can convert polyester degradation enzymes into amide hydrolases by providing effective transition state stability [199]. In addition, there are also reports that acyltransferase from Aspergillus melleus can hydrolyze amide bonds in PA fabrics [200].

Although the amide bond of PA is the same as the amide bond between amino acids that make up proteins, PA is far less sensitive to biodegradation than proteins. This may be related to PA crystallization. The presence of hydrogen bonds between PA chains increases the crystallinity of PA, which may be an obstacle to PA biodegradation [201].

## 4. Prospects

As the production of polyamides continues to increase, the environmental pollution caused by polyamide products troubles people. The disadvantages of chemical recycling degradation are obvious, and all kinds of degradation methods have unacceptable disadvantages, such as serious pollution in the production process of chemical reagents, high energy consumption, and cumbersome recovery or separation processes [202]. Finding enzymes that can degrade high-molecular-weight polyamides and exploring the recovery of polyamides under mild conditions may solve the pollution problem of polyamide materials that have already been produced. Monomers PA-6 and PA-66 can be biodegraded, so there are also some studies that synthesize environmentally degradable polyamides through copolymerization or molecular design, which also provides us with new research ideas. However, most people focus on the recycling or degradation of polyamides and may neglect the research into renewable polyamides.

### 4.1. Find Enzymes That Can Degrade High-Molecular-Weight Polyamides

Although polyamide 4 has been proven to be degradable in seawater, organisms, and soil in the literature, no enzymes that can degrade currently used high-molecular-weight polyamides have been found yet [52,53,54,55]. The main difficulty of this method lies in how to reduce the crystallinity of PA and improve the thermal stability of the enzyme (the glass transition temperature of PA is relatively high). At present, the Open Plastics project supported by Queen’s University, Ontario, Canada, is looking for new microbes and new enzymes that can degrade PA, providing new opportunities for developing effective PA degradation processes [203]. There are already studies that have achieved certain results by physically destroying polyamides by grinding and treating polyamides with enzyme combinations containing manganese peroxidase, protease, lipase, keratinase and Bacillus protease [204].

### 4.2. Explore Methods for Recycling Polyamides under Mild Conditions

Various methods for degrading polyamides have been detailed in Section 2 of this article. But perhaps due to the crystallinity and hydrogen bonding of PA, the conditions for chemically degrading and recovering PA are very severe. As a typical thermoplastic plastic, the mechanical properties of recycled polymers will be affected, leading to low-value materials. If these problems can be solved, these PA materials may not enter landfills or environments [205]. From an economic and environmental perspective, there is an urgent need for a method that allows polyamides to be fully recovered or degraded under mild external conditions.

### 4.3. Synthesize Degradable Polyamides in Natural Environments through Copolymerization and Molecular Design

By adding glycine and ester groups to the polymer backbone, synthesized poly(glycyl-e-aminocaproic acid) (nylon 26) and poly(glycyliminohexamethyleneimino-adipoyl) (nylon 266) can both be degraded by fungi [206]. Introducing substituents such as a benzyl group, hydroxyl group, and methyl group into polyamide can also improve the biodegradability of polyamide [207].

### 4.4. Degradable Bio-Based Polyamides

The raw materials for the currently produced polyamides are mostly obtained through petrochemical resources, and the petrochemical resources on the earth are very limited. It is also a viable method to develop and utilize polyamide monomers that can be produced by organisms. The biomass raw materials that can be directly obtained after chemical treatment include starch, cellulose, lignin, etc. Microbial fermentation and direct microbial polymerization can also prepare polymers such as polyhydroxyalkanoates (PHA) [83,208]. Natural high polymers that can be extracted from plants and some microbial metabolites, such as branches, straw, etc., can also become biomass resources. After the above substances are decomposed by microorganisms, many five-carbon or six-carbon sugar organic substances can be obtained, which can be used as raw materials for polymerization reactions. These substances can be treated by chemical means to obtain raw materials such as azelaic acid, decanedioic acid, 1,4-butanedioic acid, glycerol, ethanol, and 1,3-propanediol [209]. Degradable bio-based polyamides may fundamentally solve the pollution problem of polyamides and rid us of the dependence of polyamides on fossil raw materials.

## 5. Conclusions

Polyamide materials are widely used due to their excellent performance, and the environmental pollution they cause is a major hidden danger. This article reviews various methods currently used to treat polyamides and divides these methods into two categories: recycling and reuse processes and environmental degradation.

Filling and landfilling are not applicable to polyamides. Due to the nature of polyamide waste itself, it is easy to decompose and produce harmful gases and liquids, causing pollution to the environment, so polyamide waste cannot be treated by filling and landfilling. Incineration of polyamides will produce polluting toxic gases such as CO and NO_X_ on the one hand, and increase costs for treating these harmful wastes. On the other hand, the energy utilization rate of incineration treatment is very low. The use of pyrolysis oil methods is limited by energy consumption and the characteristics of the final product. Mechanical recycling has the advantages of low processing cost, less residue, and low energy consumption but also has disadvantages such as producing pollutants and deteriorating product performance. Physical recycling methods do not significantly change the performance of polyamide products, but a large amount of chemical reagents are required during processing, which will also cause pollution during preparation.

The currently reported chemical recycling methods for polyamides include hydrothermal reaction, microwave-assisted hydrolysis, acidic hydrolysis, alkaline hydrolysis, alcoholysis, hydrogenolysis, depolymerization reaction in ionic liquids, ammonolysis, etc. Generally speaking, polyamides are difficult to hydrolyze, but after heating and pressurizing polyamides can also quickly degrade in water. When the temperature reaches 302–400 °C and the pressure reaches 35 MPa, PA-6 can also degrade into α-caprolactam. Microwave-assisted hydrolysis (200 °C and 1.25 HCl/amide molar ratio) PA-66 can be completely converted into constituent monomers in 10 min. The consumption of HCl and energy consumption limit the large-scale application of microwave-assisted hydrolysis. Polyamide materials have strong alkali resistance. Alkaline hydrolysis only occurs when pH is greater than 13. Although polyamides have good acid resistance, they also degrade significantly when pH < 2 at 90 °C. The method of pyrolysis under alkaline conditions has achieved good results in recycling PA-6 monomers, but using NaOH and KOH as catalysts and conditions at 350 °C greatly increases the recycling cost. Ammonolysis is degraded into monomers and oligomers with terminal amino groups under ammonia gas, hydrogen gas atmosphere, and catalyst at 200 °C. Polyamide alcoholysis degrades into monomer derivatives under supercritical conditions and methanol at 300–370 °C. Polyamide hydrogenolysis forms alcohols and amines under ruthenium chelating catalysts toluene and 10 bar hydrogen at 100 °C. Polyamide depolymerization reaction in ionic liquids using N-methylpyrrolidone as a solvent with DMAP (4-dimethylaminopyridine) as a catalyst can degrade into monomers at 300 °C. Although there are many chemical recycling methods for polyamides, they can only be achieved under relatively harsh or strong chemical reagent conditions.

Thermal oxidation photooxidation degradation in weather degradation can only cause performance degradation of polyamide materials. After degradation, the materials will still pollute the environment and cannot solve the environmental pollution problem of polyamides. Some microorganisms in nature can degrade polyamide materials, but these environments are very rare, making it difficult to treat large amounts of polyamide waste on a large scale. Enzymes that have been reported to degrade polyamides can only act on the surface of polyamides, and can only degrade low-molecular-weight compounds.

In terms of environmental pollution, the treatment of polyamide waste should first consider reuse, followed by biological, chemical, and physical recycling, followed by energy recovery and thermal recycling, and finally consider burying in the soil. Regarding the future development trend of polyamide materials, finding enzymes that can degrade high-molecular-weight polyamides, exploring the recycling methods of polyamides under mild conditions, preparing polyamides that can be degraded in the natural environment through copolymerization and molecular design, and ultimately preparing degradable bio-based polyamides may be the final destination for polyamides.

## Figures and Tables

**Table 1:** Overview of PA mechanical recycling research [78]. Abbreviations: ABS—acrylonitrile-butadiene-styrene; CF—carbon fibers; EXT—extrusion; GF: glass fibers; HDPE—high-density polyethylene; INJ—injection molding; LDPE—low-density polyethylene; LLDPE—linear low-density polyethylene; MAPE—maleic anhydride grafted polyethylene; PA—polyamide; PAN—polyacrylonitrile; PET—polyethylene terephthalate; PMMA—polymethyl methacrylate; PP—polypropylene; PTFE—polytetrafluoroethylene; PU—polyurethane.

PA Grade	Other Materials	Processing Method	Reference
PA-6, PA-66	ABS, PC, PMMA	EXT, INJ	[84]
PA-66	PAN	INJ	[85]
PA-66	GF	INJ	[86]
PA-66	GF	INJ	[87]
PA-66	GF	INJ	[88]
PA	RUBBER	EXT	[89]
PA-6, PA-66	RUBBER	-	[84]
PA-66	GF	INJ	[21]
PA-6	GF	EXT, INJ	[90]
PA-6	ABS, PC, PET, PP, PTFE	-	[91]
PA-6	PTFE, PU	-	[92]
PA-6	LDPE, MAPE	EXT, INJ	[93]
PA-6	LDPE, MAPE	EXT, INJ	[94]
PA-6	PP, CHALK	EXT, INJ	[95]
PA-66	GF	INJ	[96]
PA-6	HDPE, LDPE, LLDPE, PP	INJ	[97]

## Data Availability

Not applicable.
